# Circulating cell-free DNA methylation profiles as noninvasive multiple sclerosis biomarkers: A proof-of-concept study

**DOI:** 10.1101/2025.02.14.25322180

**Published:** 2025-02-20

**Authors:** Hailu Fu, Kevin Huang, Wen Zhu, Lili Zhang, Ravi Bandaru, Li Wang, Yaping Liu, Zongqi Xia

**Affiliations:** 1 Department of Biochemistry and Molecular Genetics, Feinberg School of Medicine, Northwestern University, Chicago, IL 60611; 2 Robert H. Lurie Comprehensive Cancer Center of Northwestern University, Chicago, IL 60611; 3 Computational Sciences, gRED, Genentech Inc. South San Francisco, CA 94080; 4 University of Pittsburgh, Pittsburgh, PA 15260

## Abstract

In multiple sclerosis (MS), there is a critical need for non-invasive biomarkers to concurrently classify disease subtypes, evaluate disability severity, and predict long-term progression. In this proof-of-concept study, we performed low-coverage whole-genome bisulfite sequencing (WGBS) on 75 plasma cell-free DNA (cfDNA) samples and assessed the clinical utility of cfDNA methylation as a single assay for distinguishing MS patients from non-MS controls, identifying MS subtypes, estimating disability severity, and predicting disease trajectories. We identified thousands of differentially methylated CpGs and hundreds of differentially methylated regions (DMRs) that significantly distinguished MS from controls, separated MS subtypes, and stratified disability severity levels. These DMRs were highly enriched in immunologically and neurologically relevant regulatory elements (*e.g.,* active promoters and enhancers) and contained motifs associated with neuronal function and T-cell differentiation. To distinguish MS subtypes and severity groups, we achieved area-under-the-curve (AUC) values ranging from 0.67 to 0.81 using DMRs and 0.70 to 0.82 using inferred tissue-of-origin patterns from cfDNA methylation, significantly outperforming benchmark neurofilament light chain (NfL) and glial fibrillary acidic protein (GFAP) in the same cohort. Finally, a linear mixed-effects model identified “prognostic regions” where baseline cfDNA methylation levels were associated with disease progression and predicted future disability severity (AUC=0.81) within a 4-year evaluation window. As we plan to generate higher-depth WGBS data and validation in independent cohorts, the present findings suggest the potential clinical utility of circulating cfDNA methylation profiles as promising noninvasive biomarkers in MS diagnosis and prognosis.

## Introduction

Multiple sclerosis (MS) is a chronic autoimmune disorder causing inflammatory demyelination and progressive neurodegeneration, affecting nearly three million people worldwide.^1,2^ Based on disease activity and trajectory patterns, people with MS (pwMS) are classified into three main subtypes: Relapsing-Remitting MS (RRMS), Primary Progressive MS (PPMS), and Secondary Progressive MS (SPMS).^1^ As the predominant subtype (~85%) during the initial presentation, people with RRMS experience distinct episodes of new or worsening neurological symptoms (*i.e.,* active relapses) followed by periods of partial improvement or complete recovery (*i.e.,* stable remissions).^3^ In contrast, people with progressive MS (PMS), including PPMS and SPMS, experience steadily worsening neurodegeneration and neurological disability from initial symptom onset.^4^ In real clinical settings, the distinction of PMS from RRMS could take years to recognize in retrospect and cause delays in effective care. While the clinical courses of pwMS can be highly variable,^5^ standard MS monitoring tools, such as magnetic resonance imaging (MRI), have limited prognostic value.^1,6^ MRI may also not be readily accessible to all patients due to the high cost, scheduling challenges, and patient discomfort. While non-invasive fluid biomarkers, such as neurofilament light chain (NfL)^7^ and glial fibrillary acidic protein (GFAP)^8^, can inform MS disease activity, they are insufficient to classify MS subtypes or predict disease progression.^9–11^ Thus, robust and non-invasive biomarkers that timely ascertain MS subtypes and predict future progression could inform optimal clinical management, including the selection of effective disease-modifying therapy.

Circulating cell-free DNA (cfDNA) in peripheral blood represents a promising non-invasive avenue for monitoring disease activity.^12–15^ Under pathological conditions, cfDNA is released not only by peripheral immune cells but also by damaged tissues undergoing apoptosis or necrosis.^16^ Previous studies reported substantial alterations in cfDNA methylation at individual promoter regions when comparing RRMS patients with healthy controls.^17–19^ Other studies in MS patients found widespread DNA methylation abnormalities in both the central nervous system (CNS) and peripheral immune cells,^20–26^ key sources of cfDNA implicated in MS pathogenesis. By integrating cfDNA methylation patterns with publicly available reference methylomes, it is feasible to estimate the cell-type composition of circulating cfDNA and infer disease-related cell death patterns, thereby simultaneously profiling the peripheral immune system and CNS.^27–31^ For instance, an increased contribution of CNS-derived cfDNA *may* indicate a progressive MS subtype, greater disability severity, faster progression, or neurodegeneration.

To demonstrate the clinical utility as MS biomarkers, it is crucial to examine the extent to which genome-wide cfDNA methylation signatures and the inferred tissue-of-origin profiles can accurately diagnose MS, differentiate among MS subtypes, assess clinical severity, and predict future disease progression using affordable methods. Here, as a proof-of-concept, we performed low-coverage (~1×) whole-genome bisulfite sequencing (WGBS) on 75 peripheral blood samples obtained from pwMS across MS subtypes and non-MS controls in a clinic-based cohort. We assessed the genome-wide differences in cfDNA methylation and its inferred tissue-of-origin between MS and controls, across MS subtypes, and between individuals with severe disability versus otherwise. Using the cfDNA methylation profiles, we applied machine learning techniques to differentiate MS patients from controls, across MS subtypes, and between disability severity levels in a held-out test set. Finally, we developed a linear mixed model to identify “prognostic regions” in which baseline cfDNA methylation is associated with subsequent disease progression after adjusting for baseline severity and other covariates.

## Results

### Differentially methylated CpGs and regions separate MS subtypes and from non-MS controls.

**Figure 1.**
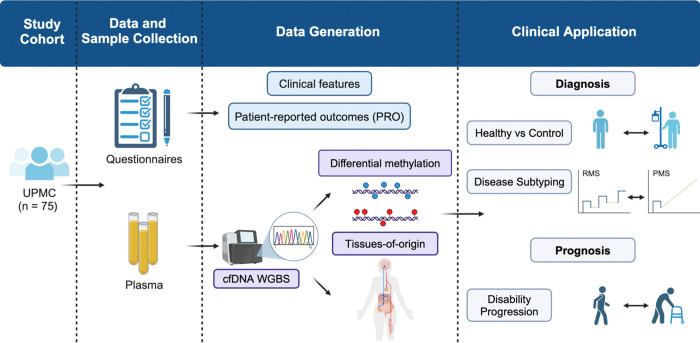
Overview of the study design.

**Figure 2.**
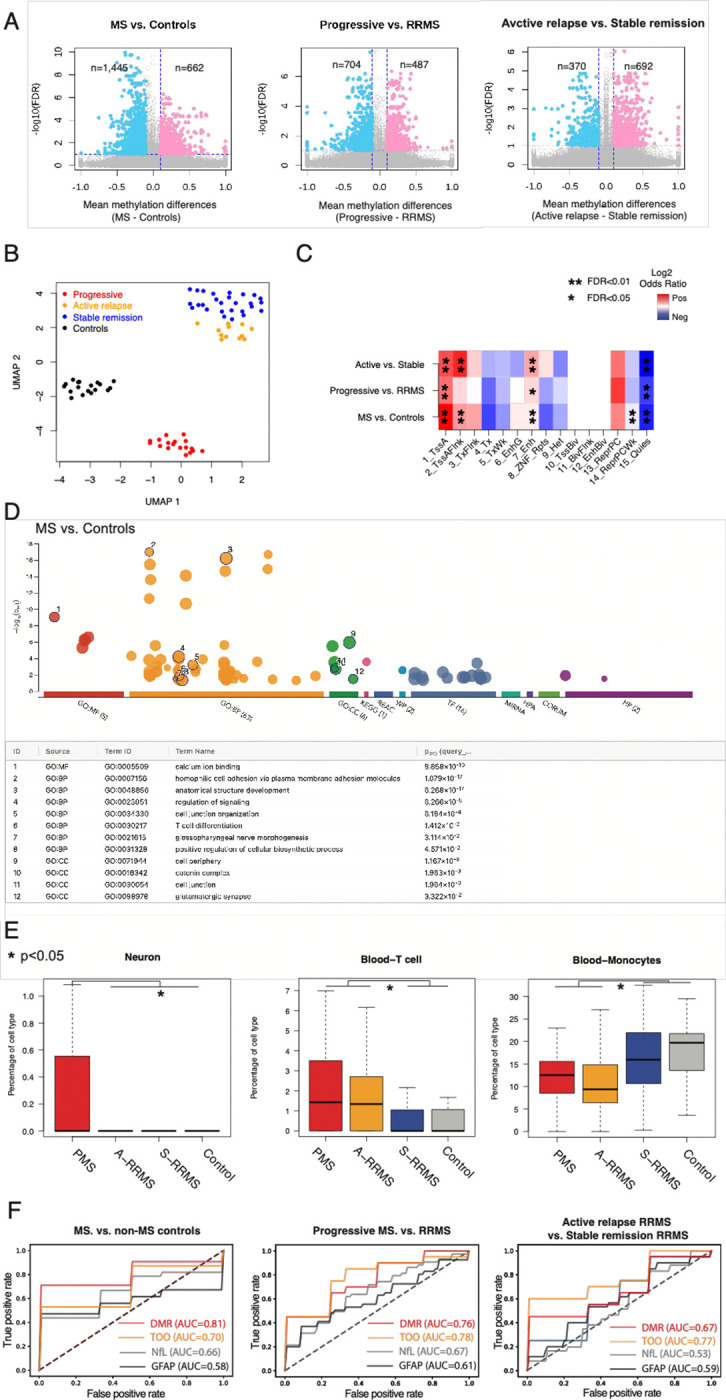
Circulating cfDNA methylation and its inferred tissue-of-origin patterns can distinguish MS from non-MS controls and among different MS subtypes. **A**. Scatter plots of −log10(FDR) vs. mean methylation differences at differentially methylated CpGs between MS and non-MS controls (left panel), between progressive MS (PMS: PPMS and SPMS) and RRMS (middle panel), and between active relapse RRMS and stable remission RRMS (right panel). Colors indicate CpGs with FDR<0.01 and absolute mean methylation difference >0.1. **B**. Uniform Manifold Approximation and Projection (UMAP) representation of cfDNA methylation level in DMRs distinguish MS patients across MS subtypes. **C**. The enrichment of DMRs in different chromHMM states (that were previously computed from GM12878 cells in the Epigenome Roadmap project). **D**. The gene ontology enrichment analysis of DMRs between MS and non-MS controls. **E**. Differences in cell death contribution from brain Neuron (left panel), T cells (middle panel), and Monocytes (right panel) across different MS subtypes (PMS, PPMS, and SPMS; A-RRMS, active relapsing RRMS; S-RRMS: stable remission RRMS) and non-MS controls. **F**. Receiver operating characteristic (ROC) curve by DMR only (red), Tissue-of-origin only (TOO, orange), NfL only (grey), and GFAP only (black) to distinguish MS and non-MS controls (left panel), progressive MS and RRMS (middle panel), and active relapse RRMS and stable remission RRMS (right panel). NfL and GFAP were measured from a subset of the patients from the same cohort as historical controls.

We collected 2 mL of plasma from 57 MS patients and 18 non-MS controls in a clinic-based prospective cohort study (*i.e.,* PROMOTE) (Table 1). Among the MS samples, 12 were obtained within 3 months of an active relapse in RRMS, 27 during stable remission in RRMS, and 18 from PMS (Fig. 1, Supplementary Table 1). We generated cfDNA WGBS data with a median of approximately 56 million paired-end reads per sample. After removing low-quality reads, we retained a median of around 34 million paired-end reads per sample, covering approximately 22 million CpGs at 1.5X per CpG (Supplementary Table 2). Using a beta-binomial regression model (RadMeth^48^), we identified significant DNA methylation changes between MS and non-MS controls as well as across MS subtypes (Fig. 2A). Specifically, we detected 662 hypermethylated and 1,445 hypomethylated CpGs in MS when compared with non-MS controls (q<0.1, absolute methylation difference >0.1). Applying the same model and thresholds, we found 487 hypermethylated and 704 hypomethylated CpGs in PMS relative to RRMS. Similarly, we observed 692 hypermethylated and 370 hypomethylated CpGs in active-relapse RRMS compared with stable-remission RRMS (Supplementary Table 3).

We next merged adjacent differentially methylated CpGs into differentially methylated regions (DMRs). This yielded 672 DMRs when comparing MS versus non-MS controls, 342 DMRs when comparing PMS versus RRMS, and 387 DMRs when comparing active-relapse RRMS versus stable-remission RRMS (Supplementary Table 4). Due to the inherent noise in methylation measurements from low-coverage WGBS, which may hinder reliable clinical correlations, we adopted a strategy previously developed for single-cell epigenetic analyses.^32,33^ We performed singular value decomposition (SVD)^34^ on the DNA methylation matrix for DMRs and selected the top components that explained the largest fraction of variability (Supplementary Fig. 1A). We then applied Uniform Manifold Approximation and Projection (UMAP)^35^ for dimensional reduction, revealing clear separation between MS and non-MS controls and among different MS subtypes (Fig. 2B).

### DMRs across MS subtypes are associated with neuronal and immune-related gene regulatory processes.

To assess the regulatory potential of these DMRs, we leveraged chromHMM states characterized in immune cells.^36,37^ All identified DMRs showed significant enrichment in active promoter and enhancer states and were markedly depleted in quiescent states (Fig. 2C, FDR<0.05, Fisher Exact test). Additionally, DMRs differentiating MS from non-MS controls and those distinguishing active-relapse from stable-remission MS were enriched at transcription start site (TSS) flanking states (FDR<0.01, Fisher Exact test), while DMRs between MS and non-MS controls showed significant depletion in weak Polycomb repressive states (FDR<0.01, Fisher Exact test).

Gene Ontology analysis further indicated that these DMRs are highly enriched in both neuronal and immune-related processes (Fig. 2D, Supplementary Fig. 2, Supplementary Table 5). For example, DMRs separating MS from non-MS controls were enriched in “anatomical structure development” and “T cell differentiation,” whereas DMRs separating active-relapse RRMS and stable-remission RRMS were enriched in “synaptic signaling.” Motif analysis revealed multiple transcription factor binding sites (TFBSs) to be significantly overrepresented in all DMR sets (Supplementary Fig. 3, Supplementary Table 5). Notably, ZBTB14, a regulator of monocyte and macrophage development,^38^ was the most enriched TFBS in DMRs, distinguishing between MS and controls. ZNF609, which modulates myoblast proliferation,^39^ was the most enriched TFBS in DMRs, distinguishing between PMS and RRMS. Finally, ZNF37A, previously implicated in myotonic dystrophy,^40^ was the top-enriched TFBS in DMRs, distinguishing active-relapse RRMS from stable-remission RRMS.

### Tissue-of-origin inferred from cfDNA methylation varied significantly across MS subtypes.

Recent studies have identified the tissue-of-origins of cfDNA molecules through their DNA methylation profiles.^27,29,31^ By deconvoluting cfDNA methylation haplotypes, we observed significantly increased neuronal cell death in the PMS patient group compared to other groups and elevated T cell death in both PMS and active-relapse RRMS groups relative to the other MS subtypes (Fig. 2E, Supplementary Fig. 4, Supplementary Table 6, one-side Mann-Whitney U test, p<0.05). These findings align with previous reports of heightened neuronal cell death in PMS and T cell-mediated pathogenesis in MS.^41^ Interestingly, we also detected markedly lower monocyte cell death in PMS and active-relapse RRMS groups, higher megakaryocyte cell death in those same groups, and reduced erythroid progenitor cell death specifically in the active-relapse RRMS group (Supplementary Fig. 4, one-side Mann-Whitney U test, p<0.05). While MS pathogenesis research has traditionally centered on T cells, emerging evidence suggests that additional cell types, including peripheral monocytes^42^ and megakaryocytes^43^, may also contribute to disease development. We interpret these results with caution as they derive from low-coverage WGBS data, which may limit the power to detect tissue-of-origin differences due to the high variability in cfDNA source estimation. Based on simulation analysis, higher-coverage (≥8×) WGBS data will be necessary to validate these observations (Supplementary Fig. 5).

### cfDNA methylation level and its inferred tissue-of-origin distinguish among MS subtypes and from controls.

Next, we examined whether DMR-based cfDNA methylation levels, together with tissue-of-origin patterns, could distinguish MS from controls and among MS subtypes. Using 10-fold cross-validation, we first identified DMRs and significantly differentiated tissue-of-origin patterns (in CNS and immune cells) within a training set. We then trained machine-learning models on these features and evaluated their performance in a held-out test set. For distinguishing MS from non-MS controls, DMRs alone achieved an area under the curve (AUC) of 0.81 (standard deviation [SD] ± 0.17), while tissue-of-origin features alone yielded AUC 0.70 (SD ± 0.19). DMRs and tissue-of-origin features outperformed neurofilament light chain (NfL, AUC: 0.66) or glial fibrillary acidic protein (GFAP, AUC: 0.58) as reported in a previous, larger study from the same clinic-based cohort as historical comparison.^44^ For distinguishing PMS from RRMS, DMRs achieved AUC 0.76 (SD ± 0.20) while tissue-of-origin features achieved AUC 0.78 (SD ± 0.21), outperforming NfL (AUC: 0.67) and GFAP (AUC: 0.61) in historical comparisons. For distinguishing active-relapse RRMS from stable-remission RRMS, DMRs achieved AUCs 0.67 (SD ± 0.29) while tissue-of-origin features achieved AUC 0.77 (SD ± 0.26), outperforming NfL (AUC: 0.53) and GFAP (AUC: 0.59) in historical comparisons. As we plan for validation in an independent dataset, these findings suggest that cfDNA methylation and its inferred tissue-of-origin are promising biomarkers for MS diagnosis and subtyping.

### cfDNA methylation level and its inferred tissue-of-origin distinguish disability severity.

**Figure 3.**
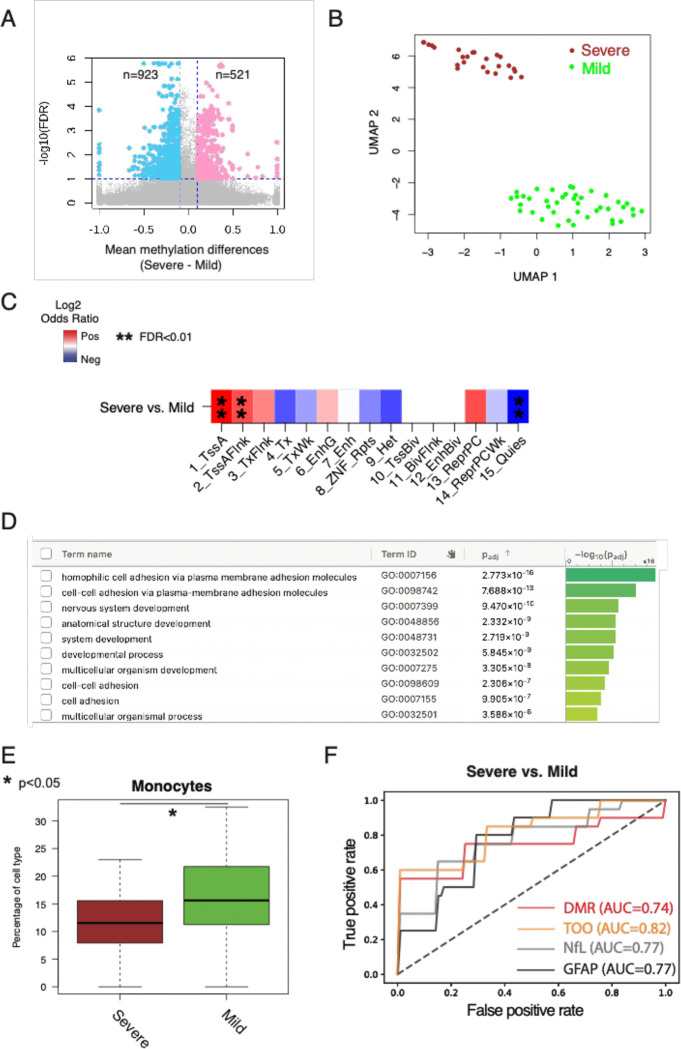
Circulating cfDNA methylation and its inferred tissue-of-origin patterns can distinguish patient-reported disability levels. **A**. Scatter plots of −log10(FDR) vs. mean methylation differences at differentially methylated CpGs between two disability severity levels based on the requirement for ambulatory assistance around sample collection: high PDDS scores (severe, PDDS≥4) and low PDDS scores (moderate/mild/normal, PDDS<4). Colors indicate CpGs with FDR<0.01 and absolute mean methylation difference >0.1. PDDS score (*i.e.,* patient determined disease steps) indicates ambulatory disability status. **B**. UMAP representation of cfDNA methylation level in DMRs can distinguish MS patients based on the binary threshold of patient-reported disability. **C**. The enrichment of DMRs in different chromHMM states (that were previously computed from GM12878 cells in the Epigenome Roadmap project). **D**. The gene ontology enrichment analysis of DMRs between MS patients with severe/moderate PDDS scores and mild/normal PDDS scores by g:profile. **E**. Differences in cell death contribution from Monocytes (left panel) and Epithelial cells (right panel) between MS patients with severe and moderate/mild/normal patient-reported disability status. **F**. Receiver operating characteristic (ROC) curve by DMR only (red), Tissue-of-origin only (TOO, orange), NfL only (grey), and GFAP only (black) to distinguish MS patients based on patient-reported disability (PDDS>5 vs. <2). NfL and GFAP were measured from a subset of the patients from the same cohort as historical controls.

We further evaluated whether cfDNA methylation profiles and tissue-of-origin patterns could distinguish MS-related disability severity level, analogous to NfL and GFAP. Using a well-validated and clinically implemented patient-reported outcome (PRO), Patient Determined Disease Steps (PDDS), we categorized MS patients in this study cohort into severe (PDDS≥4) versus normal-mild-moderate (PDDS<4) disability groups based on the requirement for ambulatory assistance around blood collection. We identified 521 hypermethylated and 923 hypomethylated CpGs in the higher disability group compared to the lower disability group (q value <0.1 and absolute methylation difference >0.1) (Fig. 3A, Supplementary Table 7). Merging adjacent differentially methylated CpGs into DMRs clearly distinguished these two disability severity groups (Fig. 3B, Supplementary Table 8, Supplementary Fig. 1B). Notably, the DMRs were highly enriched in active promoter and TSS-flanking regions and depleted in the quiescent regions (Fig. 3C), indicating strong gene-regulatory potential. Gene Ontology analysis identified significant enrichment of neuronal processes, *e.g.,* nervous system development and anatomical structure development (Fig. 3D, Supplementary Fig. 6, Supplementary Table 9). In addition, motif analysis revealed substantial overrepresentation of multiple transcription factor binding sites, including members of the AP-2 family previously implicated in cranial neural crest cell development^45^ (Supplementary Fig. 7, Supplementary Table 9). Comparing tissue-of-origin patterns between the severe and less severe disability groups demonstrated notably lower monocyte cell death and significant variability in epithelial cell death (Fig. 3E, Supplementary Fig. 8, Supplementary Table 6, one-side Mann-Whitney U test, p<0.05). These observations corroborate prior studies linking peripheral blood monocyte counts to MS severity^46^ and suggesting a potential role for epithelial cells in disease progression.^47^

To evaluate the predictive performance of distinguishing severe from less severe disability status, we used 10-fold cross-validation. First, we identified DMRs and differentially inferred tissue-of-origin patterns (in immune and epithelial cells) in each training fold. Then, we applied machine learning models for evaluation using the corresponding held-out test fold. cfDNA methylation DMRs alone achieved AUC 0.74 (SD ± 0.34), while tissue-of-origin patterns alone yielded AUC 0.82 (SD ± 0.23) (Fig. 3F). Both approaches performed comparably to historical comparisons with NfL and GFAP (AUC: 0.77 each) from the same cohort, suggesting that cfDNA methylation and its inferred tissue-of-origin can accurately assess the severity of MS disease at the time of blood draw.

### cfDNA methylation level at prognostic regions can predict future disability progression.

**Figure 4.**
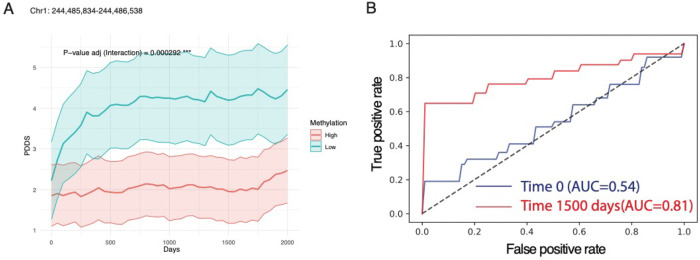
Baseline circulating DNA methylation profiles are informative of future disability severity trajectory in MS patients. **A**. Representative plots for the patient-reported disability severity (*i.e.,* PDDS) over time stratified according to baseline cfDNA methylation level (high, ≥median, red; vs. low, <median, green) at one of the prognostic genomic regions. The subgroup with higher methylation levels showed increases in patient-reported disability severity over time. The p-value has been corrected for multiple testing. The shaded area represents the 95% confidence interval. **B**. ROC curve by using the baseline cfDNA methylation level at prognostic regions to predict the binary status (PDDS>5 vs. <2) of patient-reported disability severity at 0 days (blue line) or 1500 days (red line) after the blood sample collection. *PDDS*, patient determined disease steps.

Finally, we evaluated whether baseline cfDNA methylation profile could predict future disability progression in MS. Using longitudinal PDDS data (Supplementary Table 10) and baseline cfDNA methylation levels, we constructed a linear mixed-effects model to estimate the interaction between time and baseline cfDNA methylation. After adjusting for covariates (age, gender, and race) and correcting for multiple hypotheses, we identified 7,096 potentially “prognostic regions” (FDR<0.01) whose baseline methylation levels were associated with the trajectory of subsequent disability severity over time (Fig. 4A, Supplementary Fig. 9, Supplementary Table 11). To assess predictive utility, we used a cross-validation approach with a gradient boosting model trained on baseline cfDNA methylation in these potentially prognostic regions as features and binary PDDS values (PDDS>5 vs. <2) as outcomes either at baseline (at sample collection) or ~1,500 days post–blood collection. Repeating this procedure 100 times, the performance was inadequate for predicting baseline disability severity (AUC: 0.54, SD ± 0.28) but strong for predicting future disability severity at ~1,500 days (AUC: 0.81, SD ± 0.19) (Fig. 4B). Taken together, these findings suggest that baseline cfDNA methylation at prognostic regions holds promise as a biomarker for forecasting long-term disability progression.

## Discussions

In this proof-of-concept study to assess the potential clinical utility of cfDNA methylation profiles as MS biomarkers, we generated low-coverage (~1×) WGBS data from plasma cfDNA in 75 samples, including common MS subtypes and non-MS controls. Using a beta-binomial regression model, we identified thousands of differentially methylated CpGs and hundreds of DMRs despite the challenges due to low-coverage sequencing. By integrating cfDNA methylation profiles at these DMRs and their inferred tissue-of-origin patterns, we effectively distinguished MS patients from non-MS controls, differentiated among common MS subtypes, and assessed association with disability severity. We further identified putative gene-regulatory functions for these DMRs and distinct tissue-of-origin patterns among patient groups. Notably, baseline cfDNA methylation profiles showed promising performance in predicting future disability trajectories within a 4-year evaluation window.

This study has several strengths and novelties. First, to our knowledge, this represents the first whole-genome investigation of plasma cfDNA methylation and its tissue-of-origin in MS and MS common subtypes. The tissue-of-origin patterns in MS subtypes and pwMS with different severity levels are not only largely concordant with previous knowledge but also crucially shed light on the potential unknown roles of other peripheral immune cells and epithelial cell types in informing MS subtypes and disease progression. Second, it demonstrates the potential of cfDNA methylation and its tissue-of-origin as biomarkers to *concurrently* diagnose MS, identify MS subtypes, evaluate disability severity, and predict long-term progression. Our preliminary evidence suggests circulating cfDNA methylation profile as a single-assay for both diagnosis and prognosis in MS outperforming other emerging non-invasive blood biomarkers such as NfL and GFAP from the same cohort. Third, we used samples collected from a real-world clinic cohort and disability outcome measure (*i.e.,* PDDS) already deployed in routine clinical care. The novel use of this longitudinal clinical data to identify prognostic regions underscores the potential of this approach to inform individualized clinical guidance to ultimately improve patient outcomes.

Our pilot study also has several limitations that inform future study design. First, we chose low-coverage WGBS as an affordable method for testing the feasibility of the eventual clinical implementation. While low-coverage WGBS is cost-effective for large cohorts, it also introduces substantial noise, reducing the power to detect DMRs and impeding robust estimation of cfDNA tissue-of-origin. Given that CNS-derived cfDNA is a relatively small fraction of total circulating cfDNA, this limitation is especially relevant for MS. Simulation analyses suggest that effective coverage of at least 8× (~10× raw coverage) would be necessary for reliable tissue-of-origin estimates in this cohort (Supplementary Fig. 5). We plan for higher coverage WGBS data generation to identify more definitive biomarkers and facilitate targeted methylation assays for clinical use.

Second, the variable MS disability progression trajectories among pwMS would require larger sample sizes and independent cohort validation to clinically establish biomarkers. Although we compared the cfDNA methylation profiling with other well-recognized MS fluid-based biomarkers (*i.e.,* NfL, GFAP) reported in the same clinic-based cohort, these comparisons did not always involve the exact same subsets of participants and should be viewed as approximate benchmarks. Future studies Incorporating multi-modal data (*e.g.*, cfDNA methylation, tissue-of-origin, other blood protein biomarkers, and standard clinical and demographic features) to train multi-class (rather than binary) classifiers could enhance predictive performance and clinical applicability.

Third, the MS samples included a greater proportion of RRMS than PMS, consistent with the broader clinical population and the clinic-based cohort. For analyses, we grouped PPMS and SPMS. There was insufficient sample size to perform analyses to differentiate PPMS from SPMS, which may have distinct cfDNA methylation patterns.

Finally, the observed methylation differences might reflect either cell-type-specific methylation changes or shifts in the proportion of cells contributing to cfDNA. Future investigation using purified cells or disease-relevant tissues (e.g., CNS and peripheral immune cells) could elucidate the underlying molecular mechanisms.

In conclusion, this proof-of-concept study underscores the potential for circulating cfDNA methylation profiles to serve as a robust and cost-effective fluid-based biomarker for MS diagnosis and prognosis. Future studies are required to validate these findings and establish the clinical utility.

## Materials and Methods

### Ethics approval

This research study was approved by the University of Pittsburgh Institutional Review Board (STUDY19080007) in accordance with the Declaration of Helsinki. All participants provided written informed consent to participate in the research.

### Clinic cohort and sample collection

Participants enrolled in a clinic-based long-term natural history cohort study (Prospective Investigation of Multiple Sclerosis in the Three Rivers Region [PROMOTE], Pittsburgh) during 2017–2021. The current study (Fig. 1) included 57 samples from adults with a neurologist-confirmed diagnosis of MS according to the 2017 McDonald criteria and 18 samples from adult non-MS controls. Clinical and demographic data were collected through a review of electronic health records. MS subtype and relapse status are determined by the treating neurologist. An acute relapse could be a clinical and/or radiologic event within 3 months of sample collection. We define clinical relapses as having new or recurrent neurological symptoms (deemed MS-relevant by clinicians) lasting persistently ≥24 hours without fever or infection (≥30 days from the onset of a preceding event). We define a radiological relapse as having either a new T1-enhancing lesion and/or a new or enlarging T2-fluid-attenuated inversion recovery (FLAIR) hyperintense lesion based on clinical radiology reports of routine brain, orbit, or spinal cord magnetic resonance imaging studies. Participants completed patient-reported outcomes (PROs), including Patient Determined Disease Steps (PDDS), via electronic or paper questionnaires. Research venous blood samples were collected during clinic visits. Plasma samples were isolated within four hours of phlebotomy by centrifugation at 2,000g for 10 minutes, followed by 15,000g for 10 minutes at room temperature, and stored at −80°C until cfDNA extraction and sequencing.

### Whole-genome bisulfite sequencing of cfDNA

cfDNA was extracted from 2mL plasma using the MagMAX^™^ Cell-Free DNA Isolation Kit (Applied Biosystems A29319) following the manufacturer’s protocol. The concentration and size distribution of cfDNA was measured by Qubit 1X dsDNA High Sensitivity kit (Invitrogen) and BioAnalyzer (Agilent). 5ng cfDNA was subjected to bisulfite conversion using the EZ-96 DNA Methylation-Gold Kit (Zymo D5008). Subsequently, cfDNA methylation libraries were prepared using the Accel-NGS^®^ Methyl-Seq DNA Library Kit (Swift 30096) and Methyl-Seq Unique Dual Indexing Kit (SWIFT 390384). To minimize batch effects, all cfDNA samples were processed in a single batch for both bisulfite conversion and library preparation. Qualified libraries were pooled and sequenced on the Illumina NovaSeq S6000 PE150 platform with a 20% spike of PhiX.

### Preprocess of whole-genome bisulfite sequencing data

Paired-end WGBS from cfDNA was processed using an internal pipeline. Based on FastQC results on the distribution of four nucleotides along the sequencing cycle, the adapter was trimmed by Trim Galore! (v0.6.0)^49^ with cutadapt (v2.1.0)^50^ and with parameters “--clip_R1 8 --clip_R2 15 --three_prime_clip_R1 5 --three_prime_clip_R2 5”. After the adapter trimming, reads were aligned to the human genome (GRCh37, human_g1k_v37.fa) by Biscuit (v0.3.16.20200420) with default parameters.^51^ PCR-duplicate reads were marked by samblaster^52^. Only high-quality reads were used for all the downstream analyses (uniquely mapped, properly paired, mapping quality score of 30 or greater, and not a PCR duplicate). The methylation level at each CpG was called by Bis-SNP (v0.90)^53^ with default parameters in bissnp_easy_usage.pl. Only CpGs at autosomes were used for downstream analyses.

### Identification of differentially methylated CpGs and DMRs from WGBS data

The beta-binomial model implemented in RADMeth^48^ was used to calculate DMRs between different subtypes of MS, between MS and non-MS controls, and between MS patients with high and low PDDS scores. Sex was utilized as a covariate in the modeling. “-bins 1:100:1” was used as parameters for command *adjust*. *dmrs* was used with -p 0.1 to merge differentially methylated CpGs nearby. A threshold of q value <0.1 and absolute methylation difference >0.1 was used to filter DMRs.

### Visualization of DMRs from WGBS data

DMRs from different MS subtypes were merged into a single location file. DNA methylation levels at these DMRs from 75 samples were calculated. Only DMRs with <80% missing data were kept for the analysis. The *impute.knn* function from *impute* package (v1.70.0) in R (v4.2.0) was used to impute the missing data with parameters of “maxp=“p”, rowmax = 0.8”. After *scale* transformation, *svd* function in R was applied to the methylation matrix. We plotted the first 50 singular values against the proportion of variance explained and chose the elbow point. For DMRs when comparing different MS subtypes, we chose the top six singular value decomposition components (SVDs). For DMRs when comparing between MS patients with high vs low PDDS scores, we chose the top four SVDs. Then, we used *umap* (v0.2.10.0) in R for dimension reduction and visualization.

### Enrichment analysis of DMRs

The 15-state chromHMM result at GM12878 from Epigenome Roadmap was used to calculate the enrichment of DMRs. The p-value was calculated against random intervals with the same chromosome and sizes using the Fisher exact test after multiple hypothesis corrections (via Benjamini-Hochberg). DMRs were lifted up to the hg38 genome by liftOver^54^ and Gene Ontology (GO) and motif enrichment were computed by g:profile.^55^

### Tissue-of-origin analysis by using cfDNA WGBS

Only high-quality reads mapped to the cell-type-specific marker regions were used for the tissue-of-origin analysis. The cell-type-specific DNA methylation markers (Atlas.U25.l4.hg19.tsv) were downloaded from https://raw.githubusercontent.com/nloyfer/UXM_deconv/main/supplemental/. The *wgbstools (v0.2.2) bam2pat* was used with default parameters to convert the bam files to the pat format.^29^ Then, *uxm deconv* was used estimate the tissue-of-origin fraction in each sample with default parameters.^29^

### WGBS coverage simulation for assessing tissue-of-origin accuracy

We merged processed bam files from non-MS control samples, filtered low-quality reads, and achieved ~20X effective coverage representing the population average. We randomly sampled from the merged bam files at different genomic coverages ranging from 0.01x to 12x and estimated tissue-of-origin fraction as mentioned above at each step. We repeated this process 100 times to build a distribution and estimate variance around the sample mean at different coverages.

### The linear mixed-effects model reveals the interaction effect between baseline cfDNA methylation level and time on the trajectory of disability severity

To evaluate the association between baseline biomarker (cfDNA methylation) levels and disability progression over time, we conducted a linear mixed-effects analysis using the *lmer* function from the *lme4* package^56^ in R (v4.2.0). The outcome variable was repeatedly assessed patient-reported disability severity (*e.g.*, PDDS), collected at multiple time points for each patient after baseline sample collection (for cfDNA isolation). Fixed effects included: (1) time (treated as a continuous variable), (2) baseline biomarker group (categorized as “High” vs. “Low” based on median cfDNA methylation levels in specific genomic regions), and (3) an interaction term (time × biomarker group) to assess potential differences in disability severity trajectories between groups. We controlled for age (continuous), gender, race, and ethnicity as additional covariates. (This study population included only one ethnicity group, *i.e.,* non-Hispanic). A random intercept for each patient accounted for repeated within-patient observations.

The model specification is as follows:

$${Severity}_{ij}=\beta_{0}+\beta_{1}\times {Time}_{ij}+\beta_{2}\times {Group}_{i}+\beta_{3}\times \left({Time}_{ij}\right.\times \left.{Group}_{i}\right)+\beta_{4}\times {Age}_{i}+\beta_{5}\times {Gender}_{i}+\beta_{6}\times {Race}_{i}+\beta_{7}\times {Ethnicity}_{i}+u_{i}+\epsilon_{ij}$$

Where:

- ${Severity}_{ij}$: Disability severity for patient i at time j.
- $\beta_{0}$: Fixed intercept.
- $\beta_{1}$: Fixed effect of time.
- $\beta_{2}$: Fixed effect of biomarker group (cfDNA methylation level).
- $\beta_{3}$: Interaction effect between time and biomarker group.
- $\beta_{4},\;\beta_{5},\;\beta_{6}$, and $\beta_{7}$ represent the effects of age, gender, race, and ethnicity, respectively.
- $u_{i}$: Random intercept for patient i.
- $\epsilon_{ij}$: Residual error term.

We used the *lmerTest* package^57^ to obtain p-values for the fixed effects using Satterthwaite’s method for approximating degrees of freedom. Model assumptions were checked by examining residual plots for homoscedasticity and normality. Statistical significance was set at a two-sided alpha level of 0.01. Adjustments for multiple comparisons were made using the Bonferroni procedure when testing multiple biomarkers to control the false discovery rate.

### PDDS interpolation

Given the pragmatic study design of the clinic-based cohort, PDDS scores were not always collected at identical time points for all participants. To address this, we performed linear interpolation to facilitate comparisons of baseline cfDNA methylation profiles with longitudinal trajectories of PDDS scores. Using the *interp1d* function from *SciPy* (v1.12.0)^58^, we interpolated each participant’s scores at 50-day intervals, covering the period from day 0 to day 2,000 after blood sample collection. For patients with observed values beyond 2,000 days, we also include the participant’s maximum observed PDDS score.

### Candidate genomic regions for the linear mixed model

We first selected genomic regions without completely missing data between MS patients with high PDDS (PDDS≥4) and low PDDS score (PDDS< 4) at baseline. We then merged regions located within 300 bp of one another using bedtools,^59^ resulting in 2,316,371 total regions. Samples exhibiting >90% missing values across these regions were excluded from subsequent analyses.

### Machine learning models: general comments

For all machine learning studies described below, we evaluated seven commonly used machine learning models: LogisticRegression with “l2” or “elasticnet,” support vector machine (SVM) with “rbf” or “linear” kernels, XGBoost, GradientBoostingClassifier, and LightGBM’s LGBMClassifier. Parameter tuning was conducted within each model. For DMR features, we tested various transformations (*e.g.*, raw input, top 2–20 SVD components, recursive feature elimination). A unified model or an ensemble approach is needed in future studies.

### Machine learning models for comparing MS vs non-MS controls

We employed a 10-fold cross-validation scheme. In each fold, 90% of the MS cases and 90% of the controls constituted the training set, while the remaining 10% served as the held-out test set. This approach ensured balanced case/control ratios in both training and test sets. Using a beta-binomial model (RadMeth), we identified differentially methylated CpGs and merged adjacent sites to form DMRs (≥3 CpGs, length <100 bp). Methylation densities were computed for each DMR. Missing data were marked as NA and processed using NumPy’s nan_to_num function. We then performed singular value decomposition (SVD) and selected the top two SVD components as input features. We trained a logistic regression model (LogisticRegression from scikit-learn, v1.1.1)^60^ with the following parameters:

“penalty=‘l2’, dual=False, tol=1e-5, C=120, fit_intercept=True, intercept_scaling=1, class_weight=‘balanced’, solver=‘lbfgs’, max_iter=1000”.

To calculate all possible differential tissue-of-origin patterns in CNS and immune cells, we conducted a one-sided Mann-Whitney U test to identify cell types (within CNS and immune cells) that differed significantly (p<0.05) between group comparisons (*e.g.*, PMS vs other subtypes, PMS + A-RRMS vs. S-RRMS + controls). Cell types meeting this criterion in any comparison were included as features. We trained a gradient boosting classifier (GradientBoostingClassifier from scikit-learn) with the following parameters:

“n_estimators=200, learning_rate=0.2, max_depth=10,

min_samples_split=2,min_samples_leaf=1,subsample=0.8,max_features=‘log2’”.

Because each participant had only one NfL or GFAP value, we applied a simple logistic regression model (C=1.0, using *StratifiedKFold* for the 10-fold cross-validation).

### Machine learning models for comparing PMS vs RRMS

We used the same 10-fold cross-validation scheme and preprocessing steps (as for the MS vs non-MS comparison). For DMRs features, instead of taking the top SVD components, we employed recursive feature elimination with cross-validation (RFECV from scikit-learn) in each fold to refine the final features (“step=1,scoring=‘accuracy’,min_features_to_select=1”). We trained a LightGBM classifier (LGBMClassifier in *lightgbm*) with the following parameters:

“n_estimators=100,learning_rate=0.1,num_leaves=31,max_depth=−1,reg_alpha=0.1,reg_lambda=0.1,subsample=0.8, colsample_bytree=0.8,min_child_samples=10”.

For differential tissue-of-origin features, given the smaller sample size of PMS relative to RRMS, we relaxed the tissue-of-origin p-value cutoff to 0.1. We again performed a one-sided Mann-Whitney U test (p<0.1) to select significantly altered cell types. We trained a SVM with the following parameters: “C=8.0, kernel=‘rbf’, probability=True”.

### Machine learning models for comparing active relapse RRMS vs stable remission RRMS

For DMR features, we retained the top three SVD components of DMR methylation levels as input. We trained an SVM model with the following parameters: “kernel=‘rbf’, probability=True,gamma=‘scale’,C=0.1”.

For differential tissue-of-origin features, given the smaller sample size of active relapse RRMS relative to stable remission RRMS, we relaxed the p-value cutoff to 0.2. We then used a logistic regression model with the following parameters: “penalty=‘l2’, dual=False, tol=0.0001, C=100, fit_intercept=True, intercept_scaling=1, class_weight=‘balanced’, solver=‘lbfgs’, max_iter=100”.

### Machine learning models to distinguish different disability severity levels

We excluded samples lacking PDDS scores, retaining 67. To ensure clear phenotypic distinction, we only used samples with PDDS <2 or >5, further reducing the dataset to 46. We applied the same 10-fold cross-validation and preprocessing strategies (as in MS vs non-MS comparisons). For DMRs features, we utilized xgboost^61^ with the following parameters: “max_depth=3, learning_rate=0.4, n_estimators=1000, class_weight=‘balanced’, objective=‘binary:logistic’,subsample=0.667”.

For differential tissue-of-origin features, we tested all immune and epithelial cell types using a p-value cutoff of 0.1, then trained a GradientBoostingClassifier with following parameters:

“n_estimators=200, learning_rate=0.15, max_depth=2, min_samples_split=2,min_samples_leaf=2,subsample=0.8,max_features=‘log2’”.

### Machine learning approach to predict the future disease severity

We included only samples with PDDS values at ≥3 distinct time points. We focused on 7,096 potentially “prognostic regions” identified by the linear mixed-effects model. At day 0 or ~1,500 days, samples with PDDS < 2 or > 5 were retained for analysis. We used a RepeatedStratifiedKFold (five folds, repeated 100 times). The GradientBoostingClassifier was trained with the following parameters:

“n_estimators=100, learning_rate=0.1, max_depth=5, min_samples_split=2,min_samples_leaf=1,subsample=0.8,max_features=‘log2’”.

### Statistics and Reproducibility

No statistical method was used to predetermine the sample size. The experiments were randomized to generate cfDNA sequencing libraries. The investigators were not blinded to allocation during experiments and outcome assessment.

## Supplementary Material

Supplement 1

Supplement 2

## Figures and Tables

**Table 1.** Meta-data of the pilot study cohort

	Control (N=18)	Actively Relapsing (N=12)	Stable Remitting (N=27)	Progressive (N=18)	Total (N=75)	p value
**Gender**						0.017
Female	9 (50.0%)	7 (58.3%)	23 (85.2%)	15 (83.3%)	54 (72.0%)	
Male	9 (50.0%)	5 (41.7%)	4 (14.8%)	3 (16.7%)	21 (28.0%)	
**Race**						0.522
African American	1 (5.6%)	3 (25.0%)	2 (7.4%)	3 (16.7%)	9 (12.0%)	
Asian	0 (0.0%)	0 (0.0%)	1 (3.7%)	0 (0.0%)	1 (1.3%)	
White	17 (94.4%)	9 (75.0%)	24 (88.9%)	15 (83.3%)	65 (86.7%)	
**Age at sample collection (yrs)**						0.053
Mean (SD)	57.333 (10.954)	41.500 (9.511)	48.333 (11.582)	61.056 (7.689)	52.453 (12.295)	
Range	27 – 70	27 – 57	28 – 71	46 – 71	27 – 71	
**PDDS at sample collection**						<0.001
Mean (SD)	0.607 (1.883)	2.375 (2.175)	2.861 (2.215)	4.991 (1.755)	2.812 (2.484)	
Range	0 – 7	0 – 5.800	0 – 7.200	1.875 – 7	0 – 7.200	
Missing	4	0	1	2	7	

Note: *PDDS*, patient determined disease steps

## Data Availability

The raw WGBS sequencing data generated in this study have been deposited in the Sequence Read Archive with controlled access from dbGaP under accession code (pending). The data are available under restricted access, and access can be obtained by contacting the Data Access Committee in dbGaP. The raw sequencing data are protected by data privacy laws. Processed DNA methylation level is available at Gene Expression Omnibus (GEO) with access ID (pending). The processed and de-identified data are available at zenodo.org (doi: https://doi.org/10.5281/zenodo.14803482). The remaining data are available within the Article, Supplementary Information, and Source Data file. The sample id in the supplementary information and source data cannot reveal the identity of the study subjects.
