# Supplementary material for: Circulating cell-free DNA methylation profiles as noninvasive multiple sclerosis biomarkers: A proof-of-concept study
Source: medRxiv. 2025 Feb 20:2025.02.14.25322180. Preprint. [Version 1] doi: 10.1101/2025.02.14.25322180 (PMC11875267; doi:10.1101/2025.02.14.25322180)
Supplement: Supplementary file 1 [file media-1.pdf]

Circulating cell-free DNA methylation profiles as noninvasive multiple sclerosis biomarkers: A proof-of-concept study.

Hailu Fu[1,2], Kevin Huang[3], Wen Zhu[4], Lili Zhang[4], Ravi Bandaru[1,2], Li Wang[1,2,#], Yaping Liu[1,2,#], Zongqi Xia[4,#]

1. Department of Biochemistry and Molecular Genetics, Feinberg School of Medicine, Northwestern University, Chicago, IL 60611
2. Robert H. Lurie Comprehensive Cancer Center of Northwestern University, Chicago, IL 60611
3. Computational Sciences, gRED, Genentech Inc. South San Francisco, CA 94080
4. University of Pittsburgh, Pittsburgh, PA 15260

# Corresponding email:
wangliove2@gmail.com (L.W.),
lyping1986@gmail.com (Y.L.),
zxia1@post.harvard.edu (Z.X.)

Supplementary Material

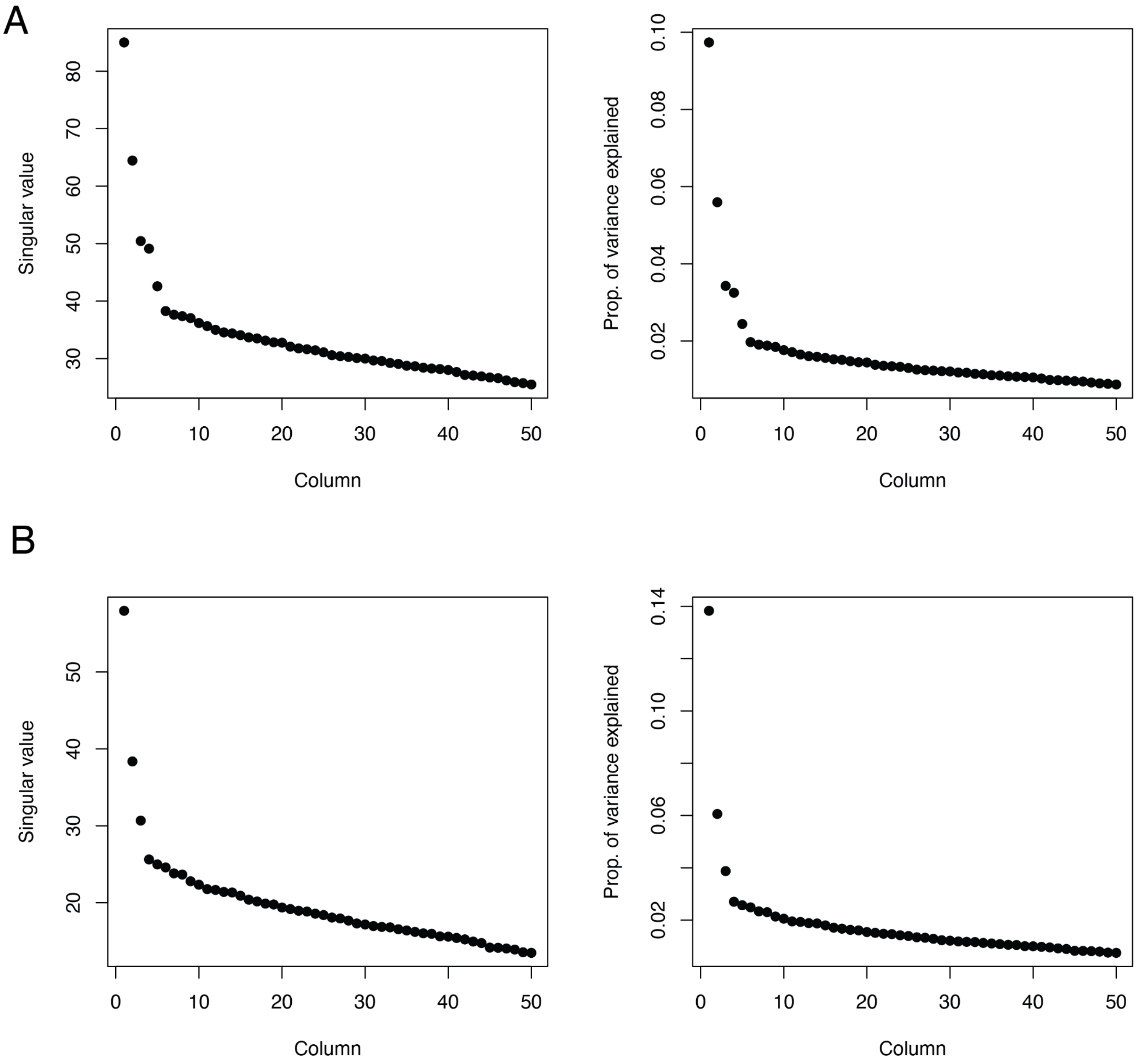

Supplementary Figure 1. Variance explained by the top singular value decompositions (SVDs).

A. Absolute (left) and proportion (right) of variance explained by the top SVDs for the cfDNA methylation level from differentially methylated regions (DMRs) that were called across MS subtypes. **B.** Absolute (left) and proportion (right) of variance explained by the top SVDs for the cfDNA methylation level from DMRs that were called between the two disability severity levels based on the requirement of ambulatory assistance around sample collection: high PDDS scores (severe, PDDS≥4) and low PDDS scores (moderate/mild/normal, PDDS<4). *PDDS*, patient determined disease steps.

A

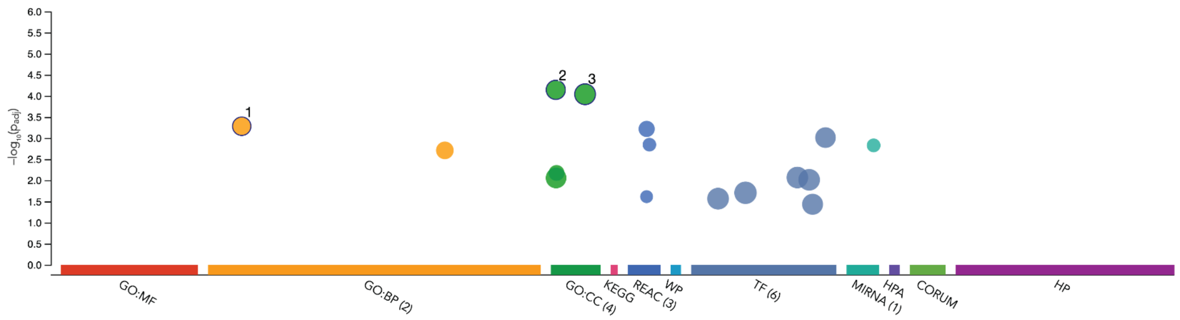

ID	Source	Term ID	Term Name	p_{adj} (query_...
1	GO:BP	GO:0007155	cell adhesion	5.228×10^{-4}
2	GO:CC	GO:0005856	cytoskeleton	7.175×10^{-5}
3	GO:CC	GO:0071944	cell periphery	9.125×10^{-5}

B

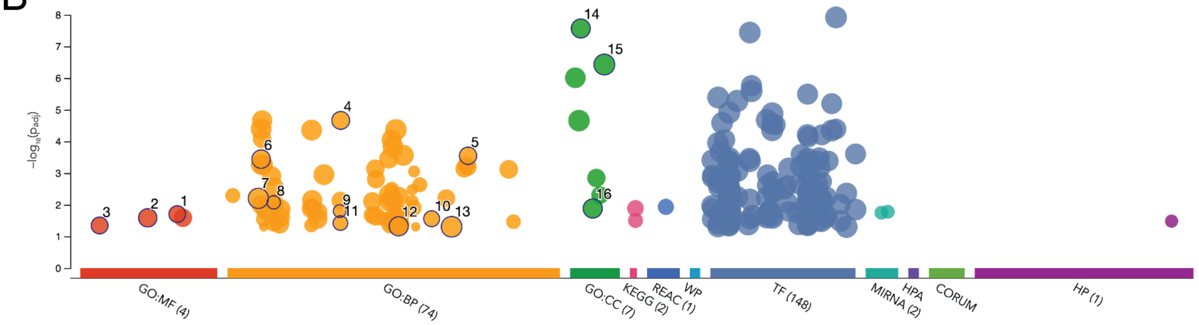

ID	Source	Term ID	Term Name	p_{adj} (query_...
1	GO:MF	GO:0050839	cell adhesion molecule binding	1.975×10^{-2}
2	GO:MF	GO:0038023	signaling receptor activity	2.592×10^{-2}
3	GO:MF	GO:0008134	transcription factor binding	4.515×10^{-2}
4	GO:BP	GO:0035295	tube development	2.174×10^{-5}
5	GO:BP	GO:0099536	synaptic signaling	2.833×10^{-4}
6	GO:BP	GO:0007155	cell adhesion	3.621×10^{-4}
7	GO:BP	GO:0006810	transport	6.285×10^{-3}
8	GO:BP	GO:0009880	embryonic pattern specification	8.415×10^{-3}
9	GO:BP	GO:0035176	social behavior	1.589×10^{-2}
10	GO:BP	GO:0070588	calcium ion transmembrane transport	2.766×10^{-2}
11	GO:BP	GO:0035265	organ growth	3.787×10^{-2}
12	GO:BP	GO:0051172	negative regulation of nitrogen compound met...	4.794×10^{-2}
13	GO:BP	GO:0080090	regulation of primary metabolic process	4.973×10^{-2}
14	GO:CC	GO:0030054	cell junction	2.721×10^{-8}
15	GO:CC	GO:0071944	cell periphery	3.738×10^{-7}
16	GO:CC	GO:0042995	cell projection	1.316×10^{-2}

Supplementary Figure 2. Gene ontology analysis on differentially methylated regions (DMRs).
A. PMS vs. RRMS. **B.** active relapse vs. stable remission in RRMS. *PMS*, progressive MS; *RRMS*, relapsing-remitting;

A

Term name	Term ID	p_{adj}	$-\log_{10}(p_{adj})$ (0 – ≤16)
Factor: ZBTB14; motif: SSCCGCGCACNS	TF:M12461	3.845×10^{-4}	
Factor: IRX2a; motif: ACRYGNNNNACRYGT; match ...	TF:M11022_1	5.134×10^{-3}	
Factor: BEN; motif: CAGCGRNV; match class: 1	TF:M01240_1	6.840×10^{-3}	
Factor: ZIC4; motif: NNCCNCCCRYNGYGN	TF:M12227	1.059×10^{-2}	
Factor: TCF-1; motif: ACATCGRGRCGCTGW; match...	TF:M11601_1	1.362×10^{-2}	
Factor: SATB2; motif: GSCGCTGTCCNNGGTGCTG...	TF:M12660_1	1.892×10^{-2}	
Factor: E2F-1:HES-7; motif: GGCRCGTGSYNNWN...	TF:M08525	1.938×10^{-2}	
Factor: RERE; motif: CNGCNSCNNGSRCRGSGSS	TF:M13129	2.030×10^{-2}	
Factor: SATB2; motif: GSCGCTGTCCNNGGTGCTGN	TF:M12660	2.039×10^{-2}	
Factor: ZNF138; motif: GCAGCRSCNSGSNCMGSG...	TF:M13144_1	2.080×10^{-2}	
Factor: ZNF432; motif: NCAGNRCCNSRGRCAGC; ...	TF:M12704_1	2.095×10^{-2}	
Factor: MAZ; motif: GGGMGGGGS; match class: 1	TF:M10432_1	2.505×10^{-2}	
Factor: BEN; motif: CAGCGRNV	TF:M01240	3.182×10^{-2}	
Factor: ZNF219; motif: SNNCAGCACCNNGGNCAG...	TF:M13149_1	3.210×10^{-2}	
Factor: ZNF142; motif: SNSCGCCCGCCCGCSCSS;...	TF:M12697_1	3.693×10^{-2}	
Factor: Egr-1; motif: NGCGTGCGY; match class: 1	TF:M04950_1	4.259×10^{-2}	

B

Term name	Term ID	p_{adj}	$-\log_{10}(p_{adj})$ (0 – ≤16)
Factor: ZNF609; motif: GNSNGGGNGCTGN; match...	TF:M13132_1	9.720×10^{-4}	
Factor: TFAP2A; motif: NGCCCYNNGGGCN; match...	TF:M04148_1	8.564×10^{-3}	
Factor: ZNF138; motif: GCAGCRSCNSGSNCMGSG...	TF:M13144	9.699×10^{-3}	
Factor: IRX-1; motif: NACRYNNNNNNNNRYGNN; m...	TF:M11018_1	1.968×10^{-2}	
Factor: ETF; motif: GVGGMGG	TF:M00695	2.712×10^{-2}	
Factor: ZNF253; motif: SNGNSCGNGGNGCKGNN; ...	TF:M13147_1	3.683×10^{-2}	

C

Term name	Term ID	p_{adj}	$-\log_{10}(p_{adj})$ (0 – ≤16)
Factor: ZNF37A; motif: CCYYGGCTCCNTSCCMN	TF:M12354	1.215×10^{-8}	
Factor: GKLF; motif: NNNRGGNGNGGSN; match cl...	TF:M07289_1	3.647×10^{-8}	
Factor: HA95; motif: CCSNSSCCNSCNCCWSCCNS...	TF:M13127_1	1.748×10^{-6}	
Factor: HA95; motif: CCSNSSCCNSCNCCWSCCNS	TF:M13127	2.588×10^{-6}	
Factor: Sp1; motif: RGGGMGGRGSNGGGG	TF:M10529	3.223×10^{-6}	
Factor: BTEB3; motif: CCNNSCCNSCCCCKCCCCC	TF:M09826	4.115×10^{-6}	
Factor: ETF; motif: CCCCGCCCCYN; match class: 1	TF:M07039_1	5.260×10^{-6}	
Factor: ZNF253; motif: SNGNSCGNGGNGCKGNN; ...	TF:M13147_1	6.433×10^{-6}	
Factor: E2F-4; motif: SNGGGCGGGAANN; match c...	TF:M09894_1	1.224×10^{-5}	
Factor: MAZ; motif: GGGMGGGGSSGGGGGGGGG...	TF:M09636	1.324×10^{-5}	

Supplementary Figure 3. Motif enrichment at differentially methylated regions (DMRs).

A. MS vs non-MS controls. **B.** PMS vs. RRMS. **C.** active relapse vs. stable remission in RRMS. Enrichment was calculated by g:profile. *PMS*, progressive MS; *RRMS*, relapsing-remitting;

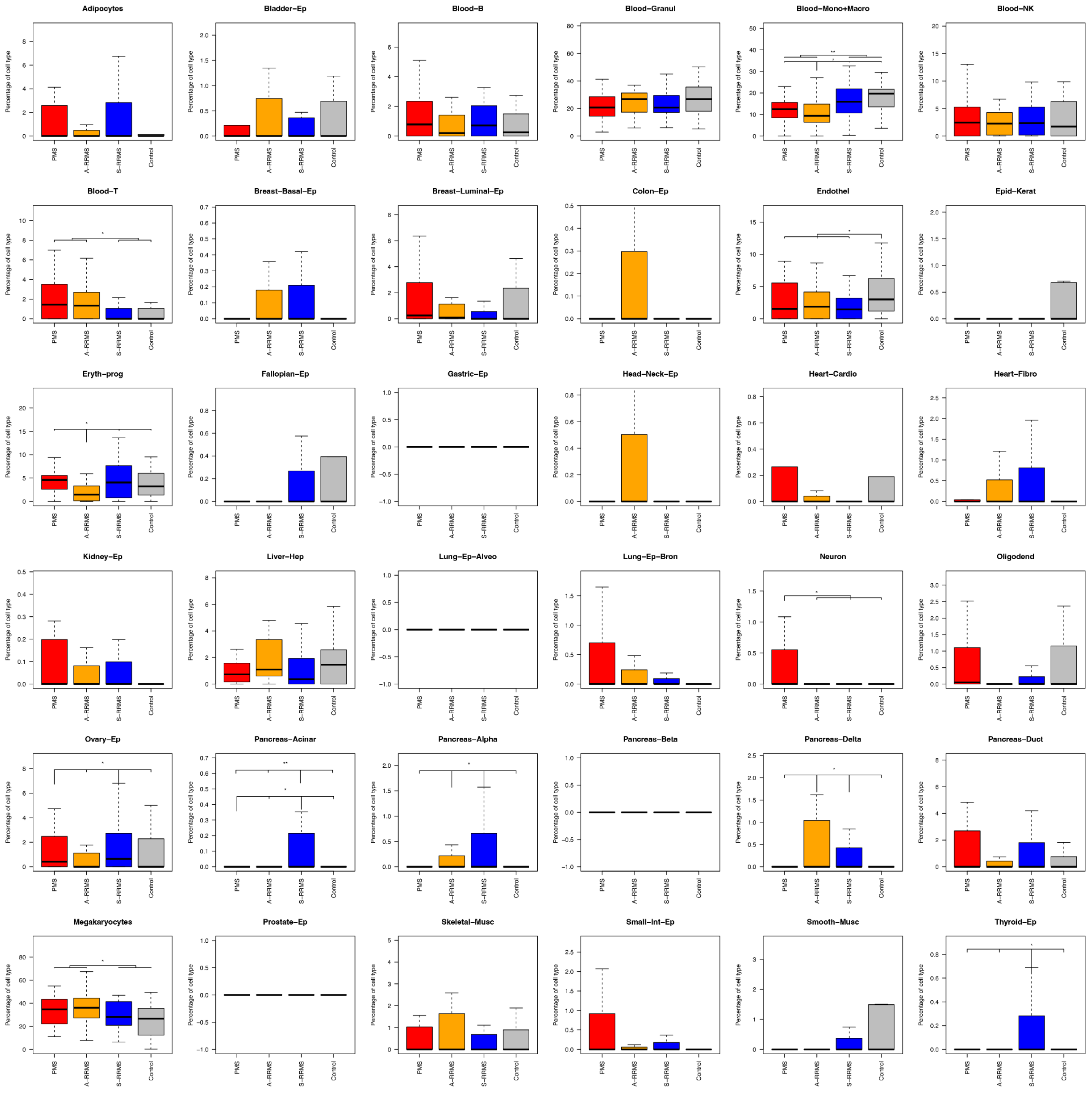

Supplementary Figure 4. Tissue-of-origin estimated from different MS subtypes and non-MS controls.

One-side Mann-Whitney U test is performed between every two groups of patients (e.g., PMS vs. A-RRMS, or PMS vs. A-RRMS+S-RRMS+Control). The side is determined if the median is larger or smaller between the groups. *A-RRMS*, active relapse RRMS; *PMS*, progressive MS; *RRMS*, relapsing-remitting; *S-RRMS*, stable remission RRMS. *, $p<0.05$; **, $p<0.01$.

A

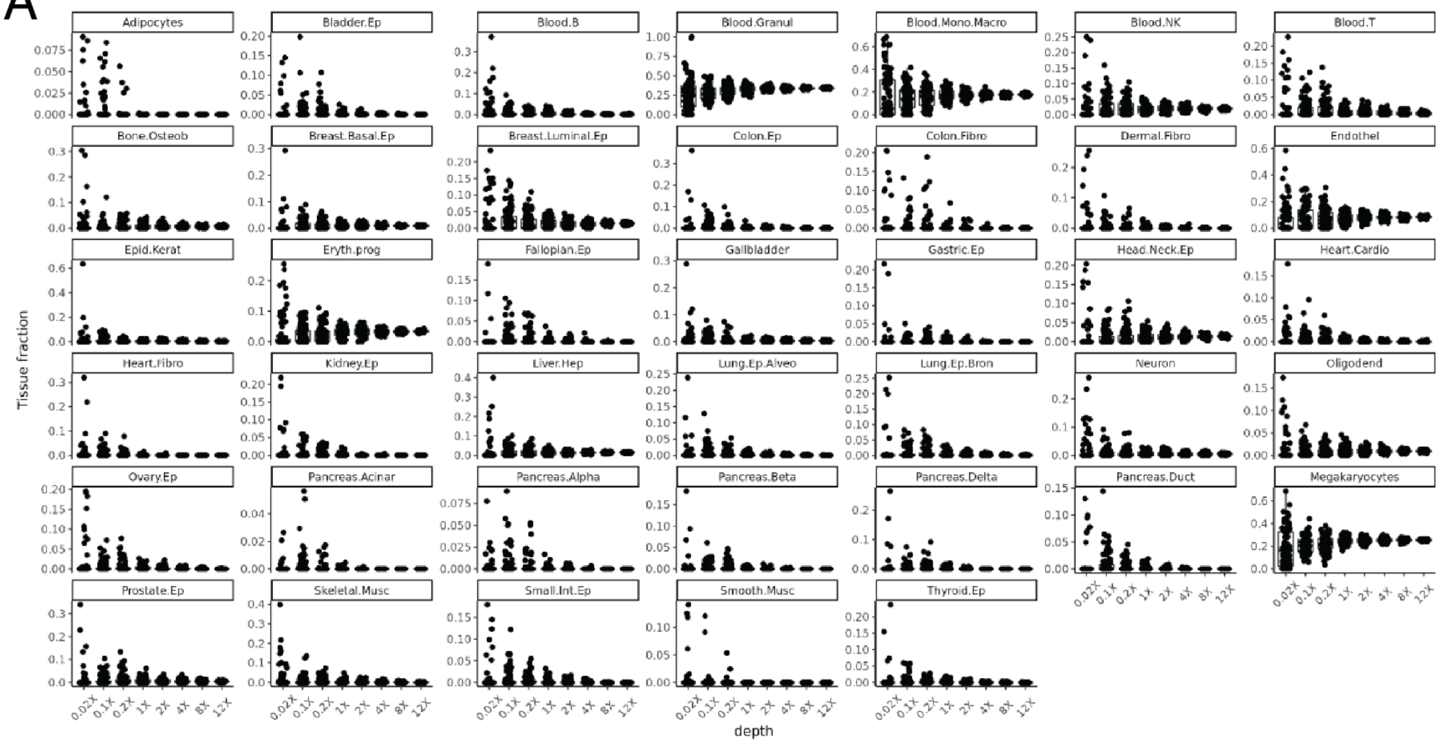
Adipocytes
Bladder.Ep
Blood.B
Blood.Granul
Blood.Mono.Macro
Blood.NK
Blood.T
Bone.Osteob
Breast.Basal.Ep
Breast.Luminal.Ep
Colon.Ep
Colon.Fibro
Dermal.Fibro
Endothel
Epid.Kerat
Eryth.prog
Fallopian.Ep
Gallbladder
Gastric.Ep
Head.Neck.Ep
Heart.Cardio
Heart.Fibro
Kidney.Ep
Liver.Hep
Lung.Ep.Alveo
Lung.Ep.Bron
Neuron
Oligodend
Ovary.Ep
Pancreas.Acinar
Pancreas.Alpha
Pancreas.Beta
Pancreas.Delta
Pancreas.Duct
Megakaryocytes
Prostate.Ep
Skeletal.Musc
Small.Int.Ep
Smooth.Musc
Thyroid.Ep
Tissue fraction
0.02X 0.1X 0.2X 1X 2X 4X 8X 12X
depth

B

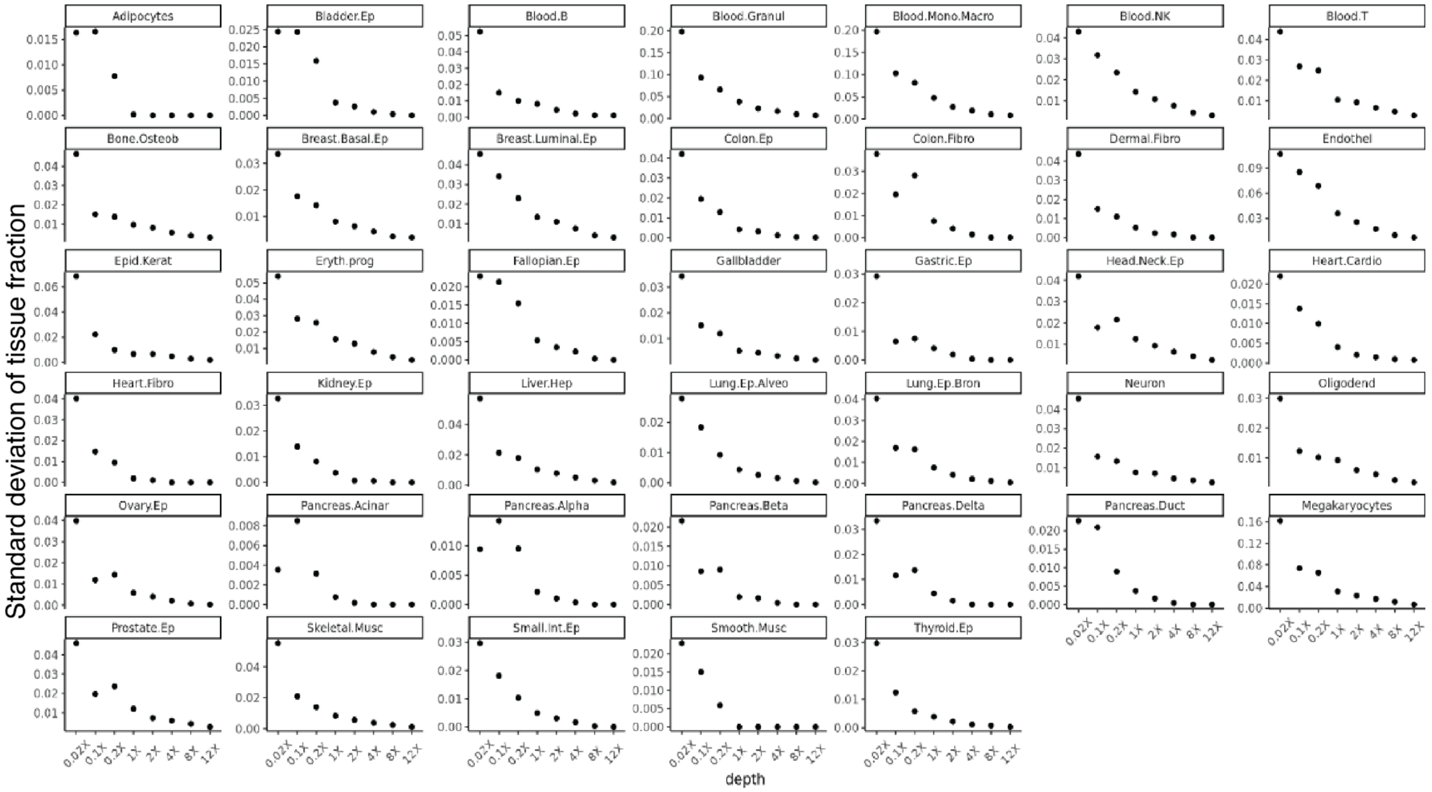
Adipocytes
Bladder.Ep
Blood.B
Blood.Granul
Blood.Mono.Macro
Blood.NK
Blood.T
Bone.Osteob
Breast.Basal.Ep
Breast.Luminal.Ep
Colon.Ep
Colon.Fibro
Dermal.Fibro
Endothel
Epid.Kerat
Eryth.prog
Fallopian.Ep
Gallbladder
Gastric.Ep
Head.Neck.Ep
Heart.Cardio
Heart.Fibro
Kidney.Ep
Liver.Hep
Lung.Ep.Alveo
Lung.Ep.Bron
Neuron
Oligodend
Ovary.Ep
Pancreas.Acinar
Pancreas.Alpha
Pancreas.Beta
Pancreas.Delta
Pancreas.Duct
Megakaryocytes
Prostate.Ep
Skeletal.Musc
Small.Int.Ep
Smooth.Musc
Thyroid.Ep
Standard deviation of tissue fraction
0.02X 0.1X 0.2X 1X 2X 4X 8X 12X
depth

Supplementary Figure 5. Simulation results on the variation of identifying tissue-of-origin at different coverages of cfDNA WGBS.

A. Tissue fractions at different downsampled coverages in different tissues and cell types. **B.** The standard deviation of tissue fraction at different downsampled coverages in different tissues and cell types.

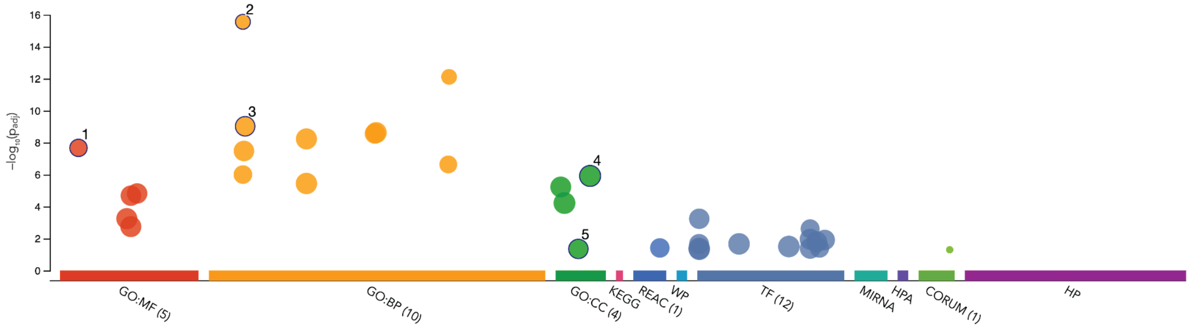

ID	Source	Term ID	Term Name	p_{adj} (query_...
1	GO:MF	GO:0005509	calcium ion binding	2.124×10^{-8}
2	GO:BP	GO:0007156	homophilic cell adhesion via plasma membrane...	2.773×10^{-16}
3	GO:BP	GO:0007399	nervous system development	9.470×10^{-10}
4	GO:CC	GO:0071944	cell periphery	1.207×10^{-6}
5	GO:CC	GO:0042995	cell projection	4.435×10^{-2}

Supplementary Figure 6. Gene ontology analysis on differentially methylated regions (DMRs) between disability severity groups.

Two disability severity groups were determined based on the requirement for ambulatory assistance around sample collection: high PDDS scores (severe, PDDS≥4) and low PDDS scores (moderate/mild/normal, PDDS<4). *PDDS*, patient determined disease steps.

Term name	Term ID	p_{adj}	$-\log_{10}(p_{adj})$ (0 – ≤16)
Factor: AP-2; motif: GSCCSCRGGCNRNRNN; matc...	TF:M00800_1	5.770×10^{-4}	
Factor: WT1; motif: NGCGGGGGGGTSMMCYN; ma...	TF:M05327_1	2.435×10^{-3}	
Factor: WT1; motif: NGCGGGGGGGTSMMCYN	TF:M05327	1.103×10^{-2}	
Factor: ZNF383; motif: SSNGGGMGGNGSNGGS; ...	TF:M12703_1	1.217×10^{-2}	
Factor: ZNF138; motif: GCAGCRSCNSGSNCMGSG...	TF:M13144_1	1.605×10^{-2}	
Factor: HA95; motif: CCSNSSCCNSCNCCWSCCNS...	TF:M13127_1	2.146×10^{-2}	
Factor: AP-2beta; motif: GCNNNGGSCNGVGGGN; ...	TF:M01858_1	2.168×10^{-2}	
Factor: RNF96; motif: BCCCGCRGCC	TF:M01199	3.134×10^{-2}	
Factor: ZNF232; motif: NCAGCASCNNGGNCAGCG...	TF:M13146_1	3.923×10^{-2}	
Factor: WT1; motif: CGCCCCCNCN; match class: 1	TF:M02036_1	4.096×10^{-2}	
Factor: AP-2gamma; motif: GCCYNCRGSN	TF:M03811	4.163×10^{-2}	
Factor: AP-2; motif: MKCCCSCNGGCG; match clas...	TF:M00189_1	4.849×10^{-2}	

Supplementary Figure 7. Motif enrichment on differentially methylated regions (DMRs) between disability severity groups.

Two disability severity groups were determined based on the requirement for ambulatory assistance around sample collection: high PDDS scores (severe, PDDS≥4) and low PDDS scores (moderate/mild/normal, PDDS<4). *PDDS*, patient determined disease steps.

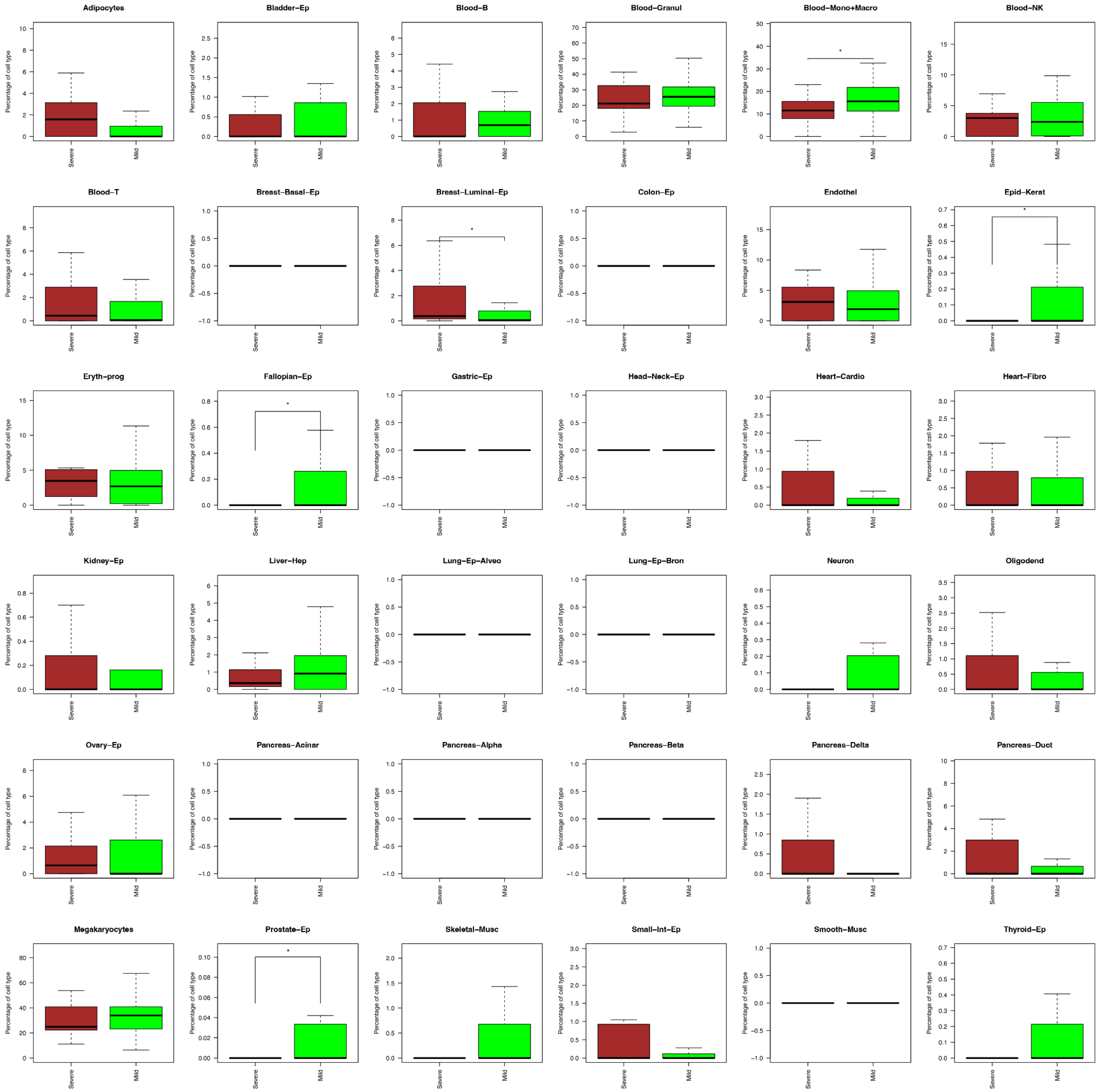

Supplementary Figure 8. Tissue-of-origin estimated between disability severity groups. Two disability severity groups were determined based on the requirement for ambulatory assistance around sample collection: high PDDS scores (severe, PDDS≥4) and low PDDS scores (moderate/mild/normal, PDDS<4). One-side Mann-Whitney U test is performed between groups of patients. The side is determined if the median is larger or smaller between the groups. *PDDS*, patient determined disease steps. *, $p<0.05$.

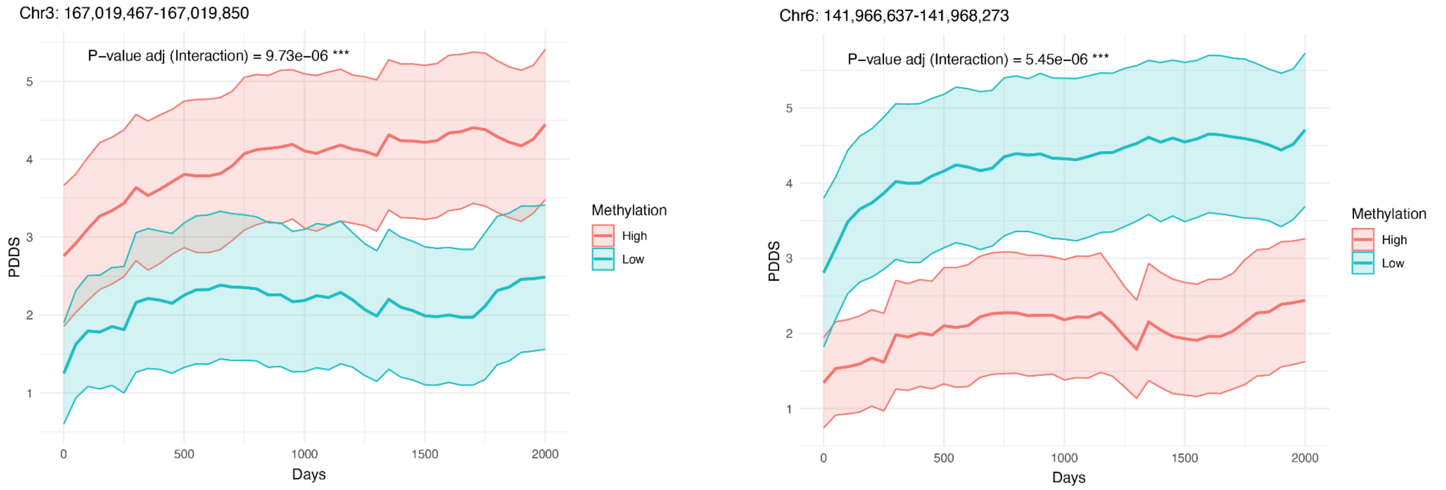

Supplementary Figure 9. Additional representative plots for the patient-reported disability severity trajectories over time stratified according to baseline cfDNA methylation level.

Baseline cfDNA methylation level (high, ≥median, red; vs. low, <median, green) at one of the prognostic genomic regions. The group with higher methylation levels (left panel) or the group with lower methylation levels (right panel) showed increases in patient-reported disability severity over time. The p-value has been corrected for multiple testing. The shaded area represents the 95% confidence interval. *PDDS*, patient determined disease steps.

Supplementary Tables.

Supplementary Table 1. Individual-level demographic and clinical meta-data.
Supplementary Table 2. Summary statistics of cfDNA WGBS.
Supplementary Table 3. Differential CpG sites in cfDNA WGBS across different subtypes of MS and non-MS controls.
Supplementary Table 4. Differentially methylated regions in cfDNA WGBS across different MS subtypes and non-MS controls.
Supplementary Table 5. Gene ontology and motif enrichment at DMRs in cfDNA WGBS across different MS subtypes and non-MS controls (from g:profile).
Supplementary Table 6. Tissue-of-origin inferences from cfDNA WGBS in each sample.
Supplementary Table 7. Differential CpG sites in cfDNA WGBS between MS patients with higher versus lower patient-reported disability.
Supplementary Table 8. DMRs in cfDNA WGBS between MS patients with higher versus lower patient-reported disability severity.
Supplementary Table 9. Gene ontology and motif enrichment at DMRs in cfDNA WGBS between MS patients with higher versus lower patient-reported disability (from g:profile).
Supplementary Table 10. Patient-reported disability measurement for each patient at different time points.
Supplementary Table 11. Genomic regions that showed differences in disability progression with different baseline cfDNA methylation level.
